# Transparency in Healthcare Reporting: The Case of External Contractors and Consultants in New Zealand’s Healthcare System

**DOI:** 10.34172/ijhpm.2021.69

**Published:** 2021-07-07

**Authors:** Adeel Akmal, Robin Gauld, Erin Penno

**Affiliations:** ^1^Centre for Health Systems and Technology, Otago Business School, University of Otago, Dunedin, New Zealand.; ^2^Department of Preventive and Social Medicine, University of Otago, Dunedin, New Zealand.

**Keywords:** Consulting Costs, External Consultants, External Contractors, Healthcare Spending

## Abstract

This study investigates the quality of reporting around the spending related to the use of external consultant and contractors in New Zealand’s 20 District Health Boards (DHBs). We make use of the publicly available annual reviews conducted by the New Zealand Parliament Health Select Committee (HSC) as well as DHB data which were retrieved using Official Information Act (OIA) requests. The quality of reporting was judged on the differences and discrepancies observed in the HSC reports each year as well as the DHB internal data. Perhaps, unsurprisingly, total spending on external consultants and contractors has been increasing over the years while the quality of reporting has been decreasing. Our analysis highlighted a number of quality issues—mistakes, discrepancies and an overall lack of standardised reporting in almost all of the DHBs. Some of these discrepancies included failure to provide information required by the HSC, differences in yearly total amounts in consecutive reports and differences between information provided to the HSC and to the authors of this article. It is hoped that this research and the prospective areas for improvement highlighted here are used as a guide to improve the quality of healthcare financial reporting.

## Background

 New Public Management (NPM) inspired reforms swept through the public sector in a range of countries from the 1980s, with New Zealand leading the way and often seen as the example to follow.^1,2^ Such reforms led to complete reorientation of the public sector, with wide-ranging implications for how public sector work is performed and by whom. A core goal was to improve efficiency of public services work and organisation. One aspect of NPM reforms was the desire to contract private providers to deliver public services. While the New Zealand and other governments backed away from NPM from the late-1990s, elements persist, including the use of external contractors and consultants (ECCs).^3^ Indeed, in many countries and areas of public work, ECC use has increased over time including in healthcare.

 ECCs provide various services—technical, policy, management expertise, service redesign and improvement, offering a different perspective, auditing services or to legitimise existing proposals for action—that a public organisation is unable to perform, deliver itself or prefers to contract out.^4^

 A recent estimate indicates global spending on ECCs providing management-related services in just the health sector has been increasing steadily,^5,6^ and is now over $6 billion annually making ECCs a highly profitable domain.^7^ Similar, increase in the reliance on ECCs in other parts of the public sector have been recorded.^8,9^ Yet the effectiveness of their services remains questionable.^9-11^ Such conclusions are mostly a result of various researchers and journalists making use of publicly available and accessible data to highlight the growing use as well as the misuse of ECCs, noting that, as a result, large sums are being transferred from the public to the private sector.^12^

 Of course, there are cases when involvement of ECCs is necessary and beneficial.^13^ However, without transparent and appropriate reporting around their use, it is impossible to even ask questions such as: whether the practice of using and relying on ECCs is excessive; if it can be reduced or needs to be reduced; whether such spending provides value for money; and, finally, if using the money to invest in building in-house expertise would be more beneficial.^10,14,15^

 As a result of the ongoing debate around these questions, health organisations, governments and policy-makers are being compelled to show that use of ECCs and their related spending is accounted for, especially in public health systems to ensure the tax money is spent appropriately.^8,16^ Like many other countries, the New Zealand healthcare system has extensively relied on ECCs to provide different types of services.^4,17^ To provide some transparency, the Health Select Committee (HSC), a standing committee of the New Zealand Parliament, conducts annual reviews of the 20 District Health Boards (DHBs), state funded organisations responsible for planning and purchasing health services for their populations, and requires DHBs to report their expenditure related to ECCs. A previous study used these reports to provide an estimate of the ECC-related spending in New Zealand between the year 2012 and 2015. However, the quality of such reporting—including the depth of detail provided and discrepancies—has not been explored. At the same time, these reports have not been collectively analysed to provide a summary of the overall spending on ECCs and the reasons for engaging them, nor to see whether the ECC-related spending have changed in any way or the quality of recorded data has improved. Building on these concerns, and the findings from an earlier study,^4^ we sought to explore the quality of reporting of ECC-related spending in the 20 DHBs by making use of HSC data and Official Information Act (OIA) requests. The findings are important as they show that ECC use has increased over time, and that there continue to be considerable concerns around the reporting of ECC expenditure. In the context of NPM, this study reveals shortcomings in the contracting model (Box 1).

**Box 1. New Zealand’s Health System**

New Zealand’s 1938 Social Security Act was the world’s first attempt to create a ‘national health service.’ Predominantly publicly-funded, Government contributes over 80% of total health funds; in 2020, this was around NZD20 billion. These funds are largely allocated to 20 geographically-based DHBs, which are responsible for planning and funding healthcare and ensuring the provision of providing health services for their respective populations. DHBs are governed by partially elected boards who act as key budget holders and decision-makers. Accordingly, DHBs have considerable strategic, management and organisational responsibilities. Among their legislative obligations, DHBs are required to seek to optimise the efficient and effective delivery of health services and to monitor the delivery and performance of services, whether they are provided by the DHB or contracted out.^18^ Funding constraints paralleled by growing demand has seen DHBs facing significant financial pressure, with almost all 20 DHBs reporting a deficit in 2020 and overspending characterised as a chronic feature of the system.^19^

------------

Abbreviation: DHB, District Health Board.

## Methods

 New Zealand has an OIA which requires public entities to release information on request unless there is a good reason for withholding it. We, therefore, used the OIA to request each of the 20 DHBs to provide details on ECCs and their use in the three study years – 2016-2017, 2017-2018, 2018-2019. Organisations are legally obliged to respond to all the OIA requests within 20 working days. A draft copy of our requests is provided as Supplementary file 1.

 ECCs were defined as parties external to DHB organisations who had been contracted to provide advice or expertise in our OIA request. We also asked DHBs whether they had a current procurement policy on the use and selection of ECCs to delve further into the managerial decisions taken to select ECCs. The OIA requests were sent for two reasons: (1) as a backup in case DHBs did not provide the required details to the HSC; and (2) to explore any variations between the data provided to the HSC and us.

 The data pertaining to ECCs and their use was imported into a spreadsheet, and classified into the categories presented in Table 1. These categories were developed after analysing the data closely.

**Table 1:** ECC Categories

**Categories**	**Example**
Service improvement	To conduct service and process improvement activities such as quality improvement initiatives.
Human resources	To conduct recruitment, training and other human resource management related functions.
Information technology	To develop and implement information technology systems.
Business management	To perform business management functions such as auditing, advisory and strategy development etc.
Others	This category contains ECCs and services that did not fall under the defined categories above. It was mostly payments to other DHBs and government ministries.
Medical services	To provide medical and clinical services. This category lists the medical and laboratory services outsourced or contracted to be delivered by the DHB.
Unknowns	This category is for ECCs whom function/reason was neither mentioned nor recognised in the annual reviews.
Miscellaneous	This category included items including buildings and development, maintenance, laundry services etc.

Abbreviations: DHB, District Health Board; ECC, external contractor and consultant.

 For a full list of entries under each of the categories, see Supplementary file 2. While the information is publicly available, we have still taken the liberty to remove the DHB names so that the research and its findings are not used to identify individual DHB organisations, and instead is focused on the study aims—documenting and improving the transparency and quality of reporting around ECCs.

 This study had a different method from its predecessor, so direct comparison of findings was not possible. Therefore, we sought to draw broad comparisons between the findings from the two studies. This comparison is provided later in the results section.

## Results

###  Total Spending on External Contractors and Consultants

 Our first findings are related to total spending by the DHBs on ECCs and the reasons for ECC use between financial years 2016 and 2019. As stated above, this was achieved by categorising individual DHB responses under the categories presented in Table 1. In cases when the reasons for recruiting ECCs were not provided, a search amongst the other DHB responses was conducted to determine whether another DHB used the same ECC, and then the purpose was replicated in the list. For example: DHBs recruited similar ECCs for construction-related work. While some DHBs explained the nature and the reason for recruiting the ECCs, the others did not. In such circumstances, the reason for recruitment for the particular ECC was replicated to other DHBs as well. Similar examples were found for service improvement, auditing, asbestos removal and many other functions. When this did not satisfy our goals, ECC names were searched for on the internet to identify the core service provided by them. In cases where the authors were unable to identify the reason for hiring particular ECCs, they were categorised as *Unknowns*. Table 2 provides details regarding the total spending on ECCs by the New Zealand DHBs in the categories mentioned earlier in the methods section.

**Table 2:** DHBs Spending on ECCs, 2016-2019

**DHBs/Services**	**Service Improvement**	**IT**	**Human Resource**	**Business Management**	**Unknown**	**Others**	**Medical Services**	**Miscellaneous**	**Total Per DHB**
DHB 1	4 589 833	393 507	11 943 369	644 640	12 096 808	1 167 174	285 248	892 305	32 012 884
DHB 2	2 581 798	747 693	1 000 723	1 202 092	1 210 329	250 852	24 734	1 956 378	8 974 600
DHB 3	2 651 844	15 390 936	3 289 981	11 450 702	-	10 404 963	13 630 960	76 516 022	133 335 408
DHB 4	3 084 866	1 119 854	641 614	3 276 610	408 578	57 602	29 369	1 779 156	10 397 649
DHB 5	1 242 997	194 435	69 316	1 540 446	900 577	1 788 805	252 311	261 809	6 250 697
DHB 6	780 676	-	142 533	31 148	-	128 444	680 036	1 826 111	3 588 947
DHB 7	795 609	303 415	81 857	238 247	5485	53 381	-	246 637	1 724 631
DHB 8	-	808 037	301 696	873 811	-	2 214 643	405 364	250 555	4 854 106
DHB 9	1 802 393	332 487	-	649 183	-	85 000	-	316 076	3 185 139
DHB 10	560 588	67 079	228 321	786 505	-	36 586	-	348 363	2 027 442
DHB 11	875 440	34 470	137 877	375 181	-	212 558	-	358 481	1 994 006
DHB 12	1 565 441	577 278	918 848	566 281	-	552 654	2000	848 169	5 030 671
DHB 13	-	-	3 212 866	-	-	29 559	15 509 204	-	18 751 629
DHB 14	50 000	-	-	40 000	-	-	-	22 375	112 375
DHB 15	1 234 438	216 634	1 259 152	1 510 534	4980	19 913	149 180	2 498 107	6 892 939
DHB 16	10 404 133	2 418 507	459 432	4 060 558	-	1 097 336	145 692 684	9 374 287	173 506 937
DHB 17	159 441	1 207 122	237 023	41 016	-	-	-	531 248	2 175 850
DHB 18	3 154 186	702 445	4 064 798	1 853 326	75 436	579 092	545 722	1 337 340	12 312 345
DHB 19	606 683	25 000	103 392	437 969	-	-	-	873 342	2 046 386
DHB 20	437 317	-	111 453	52 908	-	7955	-	-	609 632
Total Per Service	36 577 682	24 538 899	28 204 251	29 631 157	14 702 193	18 686 516	177 206 812	100 236 763	429 606 262

Abbreviations: DHB, District Health Board; ECCs, external contractors and consultants; IT, information technology.

 As illustrated, the largest ECC expenditure category was for various medical services which accounted for over NZD177 million. According to the analysed data, these are the medical, laboratory and clinical services DHBs have outsourced to private contractors—both individual contractors and organisations—permanently or temporarily.

 This figure is likely to be higher as eight DHBs did not report any expenditure for medical services and delivery. Following that, service improvement and management-related recruitment of ECCs cost over NZD36 million followed by business management (NZD29 million), human resources (NZD28 million) and information technology (NZD24 million). It should be noted that there is some overlap between the spending categories. For example, training and development was labelled as human resource-related expenditure even when training was often focused on service improvement or information technology systems. Similarly, audits were always categorised under business management services, even when these audits were related to any of the other ECC services. In total, DHBs spent over NZD152 million on ECCs, excluding *medical services *and expenditure categorised as* not included*, with spending increasing steadily over the years data were collected on. The inclusion of these two categories brings total expenditure up to over NZD429 million over the course of three years.

###  Quality of Reporting

 Our second finding relates to the quality of reporting and the discrepancies across year-on-year reporting periods identified within HSC annual reviews where DHBs are asked to provide information on their use of ECCs (Box 2).

**Box 2. HSC Question Pertaining to the Use of ECCs**

How many contractors, consultants, including those providing professional services, were engaged or employed in 2016/2017 and what was the estimated total cost? How did this compare to each of the previous four financial years, both in terms of the number engaged and the total cost? For each consultant or contractor that has been engaged in the previous four financial years please provide the following details:

- Name of consultant or contractor
- Type of service generally provided by the consultant or contractor
- Details of the specific consultancy or contract
- Budgeted and/or actual cost
- Maximum hourly and daily rates charged
- Date of the contract
- Date the work commenced
- Completion date
- Whether tenders were invited; if so, how many were received

Whether there are proposals for further or following work from the original consultancy; if so, the details of this work?

------------

Abbreviations: HSC, Health Select Committee; ECCs, external contractors and consultants.

In many cases, DHBs did not respond to the HSC as required. Some DHBs did not provide reasons for using consultancies or type of consultancy procured. Similarly, questions around the date of contract, budgets, starting/finishing date of the consultancy as well as information about inviting tenders were mostly missing. In addition, one DHB failed to provide data for one financial year in question, while another provided HSC with password protected copies of their annual reviews—that were uploaded on HSC website—meaning the data were not publicly accessible. This was not an isolated event; we found that some of the documents requested by the HSC were unavailable on their website over multiple years. In our quest to find these documents, support staff from the HSC explained that they had repeatedly asked DHBs to submit files without password protection which had not been complied with.

 A follow-up question in our OIA request concerning individual DHB procurement policies for ECCs suggested that around seven DHBs either did not have any procurement policy or they had a partially developed procurement policy that did not cover all of the aspects required to make decisions around using ECCs. Similarly, only three DHBs defined what they considered as ECCs, and what kind of services would be listed in the analysed documents. Consequently, some DHBs failed to provide the total amount of money spent on ECCs every year while some DHBs failed to provide information regarding the use of ECCs in the last four consecutive years. Even when DHBs provided such information, the sum totals of these consecutive years were mostly different in at least eight of the DHBs.

 As mentioned earlier, a separate OIA request was also sent to DHBs requesting they provide details about the use of ECCs. These details were compared with those provided to HSC. 19 (out of 20) DHBs replied, 13 provided detailed answers while six DHBs referred us to HSC documents. However, although acknowledging the request, one DHB provided no further response. As they are legally obliged to provide the requested information (or provide a reason why it cannot be provided), by not providing it, they could be reported to the office of the National Ombudsman. However, we decided to not pursue this and elected to use the data provided by the respective DHB to the HSC.

 A cross-comparison of DHB responses to HSC with their response to us concluded that 12 DHBs provided us with different answers from the HSC even when we had asked for similar information. In most cases, the differences were substantial. This included differently declared amounts spent on ECCs per year and different numbers of ECCs per year. Such problems not only illustrate the weak reporting around ECCs, but also the poor compliance with OIA requests, and seemingly, HSC requests. The points summarised above are presented in Table 3.

**Table 3:** Discrepancies in Year to Year Reporting of External Consultancies to the Health Select Committee

**DHBs**	**Reasons Provided for Consultancies**	**Yearly Sums Discrepancies **	**Provide Definition of ECCs**	**Policy for ECCs**	**Difference Between OIA Requests and HSC Reviews**	**Other Issues**
DHB 1	No	Difference found	No	Yes	Did not provide answers in OIA and referred us to HSC review	No mention of tenders, start date/end date
DHB 2	Yes	No difference found	No	Yes	Did not provide answers in OIA	No mention of tenders, start date/end date
DHB 3	Yes	Previous year totals not found	No	Yes	Did not provide answers in OIA and referred us to HSC review	No mention of tenders, start date/end date, only details of accounts which charged >$50 000 provided
DHB 4	Yes	Difference found	No	No, the delegation for using consultants currently sits with the CEO	Yes	Provided full details for 2016/2017; but details missing for subsequent years. 17/18 tenders, start date etc/18/19 tender details missing
DHB 5	No	Difference found	Yes	Yes	Difference in totals, difference in number of consultants	No mention of tenders, start date/end date
DHB 6	Yes	Info not provided	No	No	Yes	The attached sheets are not aligned. It is because of the.xlsx being imported to.docx
DHB 7	Yes	No difference found	No	Yes, policy issued in 2004, no major changes since 2006	Acknowledged OIA, but never replied	Full details for 2016/2017; but tender details missing in subsequent years
DHB 8	Yes	No difference found	No	Yes	Yes	Only the 2017/2018 file accessible. Data for other years password protected
DHB 9	No	No difference found	Partial	Yes	Yes	OIA response provided only some details, no tenders, no start date etc. HSC files included total spend only
DHB 10	Yes	Difference found	No	Yes	No, they provided HSC files in OIA	Reasons for engaging ECCs vague, no start/finish dates
DHB 11	Yes	No difference found	Partial	Yes	Yes	No mention of tenders, start date/end date
DHB 12	Yes	Difference found	No	No	Yes	No mention of tenders, start date/end date
DHB 13	Yes*	Info not provided	No	No	Yes	No mention of tenders, start date/end date. Previous years’ records are not provided as asked by HSC
DHB 14	Yes	No difference found	No	Yes	Yes	None
DHB 15	Yes*	No difference found	No	Partial	Yes, OIA had only 17/18 and 16/17	No mention of tenders, start date/end date. Clinical and building contractors were not included
DHB 16	Yes	Difference found	No	Yes	Did not provide answers in OIA	No mention of tenders, start date/end date
DHB 17	Yes	Difference found	No	No	Yes	No mention of tenders, start date/end date
DHB 18	Yes	Difference found	No	Yes	Yes	16/17 missing; No mention of tenders, start date/end date
DHB 19	Yes	Info not provided	No	Yes	Did not provide answers in OIA	No mention of tenders, start date/end date
DHB 20	Yes	No difference found	No	Partial	Yes	NA

Abbreviations: DHB, District Health Board; HSC, Health Select Committee; ECCs, external contractors and consultants; OIA, Official Information Act; CEO, chief executive officer. Note: Symbol * stands for partially provided information pertaining to recruiting consultancies.

###  Comparison Between 2016 and 2019

 A comparison between a prior research analysing New Zealand DHB spending on consultants and contractors between the years 2012 and 2015 and this research had the following findings. After almost five years:

- DHB spending had increased substantially based on the data provided in the earlier research, from an upward level of around $60 million annually in 2016 to well over $160 million annually in 2019.
- Some DHBs still did not have policies around the use and recruitment of contractors and consultants.
- Some DHB annual reports were still missing or inaccessible on the HSC website.
- Issues around compliance with the OIA continue, with some DHBs providing only limited or partial responses, or referring the authors back to HSC annual reviews, which were also often incomplete or showed inconsistencies year to year.
- There continues to be no uniform or standardised definition of contractors and consultants. Hence, DHBs tended to report these hires very differently from one another.
- DHBs still tended to pick and choose the type of ECCs they reported upon. For example, some DHBs reported ECCs related to medical services delivery while others ignored them completely.

## Discussion

 Transparent healthcare reporting is one of the most significant strategies to control healthcare spending and improve public opinion about the healthcare system.^20^ In New Zealand, the statutory HSC, an organ of Parliament and composed of its elected members, is intended to bring such transparency. However, our analysis indicates that healthcare expenditure on ECCs has increased around 150% from $60 million in 2016 to $160 million in 2019.^4^ Our study revealed that unclear procurement policies and procedures, inconsistent and generally inadequate reporting depicts nothing has changed since our prior evaluation. Despite New Zealand’s freedom of information legislation, healthcare reporting on ECCs appeared to lack transparency and justification to government bodies or any consistency in terms of public reporting. Notably, we found an absence of clear national procurement and reporting policy nor, where this did exist, application of and adherence to this. Arguably, this situation is a source of considerable duplication with corresponding waste of public funds. Indeed, ECCs are estimated to cost twice as much as equivalent permanent employees.^21^

 It has been posited that the increased reliance on ECCs in health systems globally is due to the adoption of NPM reforms from the 1980s onwards.^7,9^ Arguably, this increased reliance requires deeper investigation. While our research has been focused on New Zealand, there are studies which highlight similar trends in the United Kingdom as well as in Australia.^6,10,11^ The reliance on ECCs appears to be a sector-wide problem, and therefore demands a comprehensive approach when being investigated. Our research is a start; further research is needed, including across other areas of the public sector which are also known to rely on ECCs.

 In New Zealand, the HSC must intervene before this reliance on ECCs makes healthcare budgets even more stretched than they currently are. To do this, HSC should start by improving the DHBs’ compliance to the required reporting standards and develop further policies that provide a better picture of the health systems’ reliance on ECCs.

 In this research, we divided the ECC spending into various categories. This was done for a number of reasons. From a systems perspective, this categorisation highlights the largest areas for in-house skill readiness and development within the healthcare system. From a policy perspective, it reflects the need to improve healthcare reporting, and also the need to develop better policies around the definition as well as the use of ECCs. Finally, from a research perspective, such data can be used as a basis for further research focusing on particular ECC categories and perhaps determine better ways to use the very large sums of money involved. According to the New Zealand Treasury,^22^ a total of NZD13.236 billion in government funding was provided to the 20 DHBs for services to meet the needs of their respective populations in 2018-2019. This means that, at NZD163.7 million, ECC spending amounted to almost 1.25% of total budget in that year. While a comparatively small amount, there remain important questions of public accountability for how these funds are spent, the gains made as a result, and whether value for public money is attained.

 Clearly, the HSC as well as New Zealand’s Ministry of Health must improve upon healthcare financial reporting, and perhaps develop a national procurement and reporting policy on ECCs. The HSC must create guidelines for the DHBs and ensure that they follow these. These guidelines should outline appropriate ECC use and reporting. There should be strict definitions of expenditure categories: the HSC could use the categories developed in this research in their reporting as a start. Providing categories would allow each DHB to define the different types of ECCs and supply guidance for how they should be reported. For example, we found that some DHBs did not consider temporary clinicians as well as medical service providers as ECCs and, hence, they did not report them, while others did. Similarly, there were instances where some DHBs reported recruitment agencies as consultants under business management or human resource categories, whereas others reported such expenses under the medical service providers category.

 For the Ministry of Health, healthcare reporting is of significant value; it not only highlights current issues, but also possible future concerns for the health system. For example, in the *service improvement* category, New Zealand’s DHBs spent more than $36 million on over 200 different consultants. At the same time, there is a Crown Agency working under the Ministry of Health responsible for service improvement with its own funding—the Health Quality and Safety Commission. Such spending at DHB-levels may suggest the Health Quality and Safety Commission role needs to be expanded so that it can assist DHBs in terms of service improvement programmes, or at least provide them with support to ensure that ECC service improvement services are audited for impact. Similarly, our analysis highlights a need for more centralisation of certain functions such as auditing and business management, human resource development, and IT services as DHBs individually spend considerable sums of money on such functions. Finally, this study indicates that the New Zealand Ministry of Health would be justified in seeking to develop a national policy for the use of ECCs.

 Having a national procurement and reporting policy which the DHBs must follow would help to minimise reporting issues highlighted in this research. It would also help reduce the allocation of public funds to activities such as service improvement when entities such as Health Quality and Safety Commission are already in existence, with a remit to support service improvements. Furthermore, the HSC should also seek explanations for various discrepancies in annual reviews highlighted by our analysis, and if and when DHBs do not respond to their question appropriately, this should be followed up on.

 We believe the findings of this study are not only crucial for the New Zealand healthcare system, but other areas of public sector in New Zealand and abroad as well. This study has probably revealed the tip of the iceberg. Further research within the healthcare and broader public sector is required to not only highlight the extent of ECC use but perhaps to also reveal best-practices that may be transferable and implementable elsewhere. Having a single procurement policy for the use of ECCs is just one example that could be identified from best practice and extended nationwide. Research such as that presented in this article could be used to inform bodies such as the HSC along with political leaders and decision-makers; it could also help with development of expertise for in-depth performance management and measurement analyses. Very importantly, the study indicates that the NPM-inspired idea that contracting is cost-effective, efficient and provides better public services is questionable.

 This study has limitations that should be highlighted. The analysis and sums presented in this article should be treated with caution as they do not capture the full picture. As noted, currently, DHBs define ECCs and their use themselves. This means some ECCs are not represented in the annual HSC reviews which were our primary source of data. Moreover, whether the use of ECCs added any value to the overall organisation or the patient care processes was not audited or analysed, and could be the subject of further research. A similar point was raised by previous research which explored the use of ECCs in NHS.^9^ Second, our analysis was based on the answer to a single question about ECCs which may not provide all details. For example, another question by the HSC requires DHBs to list all the ECCs contracted by DHBs for over NZD1 million. We did not include this because almost all such ECCs were either medical providers, insurers or construction developers.

## Conclusion

 This research depicts the New Zealand healthcare system’s reliance on ECCs, estimates related expenditure and the processes around procuring ECCs and reporting on ECC expenditure. It revealed that ECC expenditure reporting is substandard and has increased over time. It is possible that such information obtained through this study could be interpreted and highlighted by the media and general public in a negative way.^9,16^ That was not the intention of this study, as negativity could lead to restricting the use of ECCs when there is a genuine need. Therefore, it is hoped that this study will lead to improved healthcare reporting as a first step towards ensuring evidence of value of ECCs.

 New Zealand could become an exemplar by developing better reporting and procurement policies for ECC use. To do this, both the HSC and the Ministry of Health would need to redesign the current reporting mechanisms, and ensure DHBs comply with requirements. Arguably, they should do so in the interests of accountability for public money and management. Unfortunately, performance reporting and measurement systems are notorious avenues for ‘gaming,’ so any redesign would need to be handled with care.^23,24^

 Finally, ECCs can encounter challenges and restrictions in ensuring their proposed solutions and implementation frameworks provide desired improvements. Further research could probe such barriers and how ECCs and their client agencies seek to overcome them along with longer-term impacts of ECC work. There has been a debate around the resistance towards NPM practices from the corporate sector in healthcare organisations,^25-28^ and investigating the perspective of ECCs might improve understanding of this debate. Our findings also have implications for other health systems. For example, we know little about ECC expenditure in OECD member countries which could usefully be investigated and subject to comparative analyses. In countries committed to open and transparent government, the methods developed for this study could be utilised.

## Acknowledgements

 We would like to thank the employees of all the New Zealand DHBs and the HSC (New Zealand Parliament) who responded to our requests and helped us access the data for this research. Also, the anonymous reviewers for their valuable comments and feedback.

 A special thanks to Natasha Podgorodnichenko, Wayne Dillon and Rebecca Zitoun for a lot of things, but mainly the endless lunchtime discussions on random issues that affect the big picture. This manuscript is perhaps in part a result of those lunch breaks.

 Finally, a very special acknowledgement for Mr. Muhammad Akmal for his love, guidance, support and everything else.

## Ethical issues

 The paper relied on publicly available data. No ethical issues were identified in the process.

## Competing interests

 Authors declare that they have no competing interests.

## Authors’ contributions

 AA collected and analysed the data. AA and RG wrote the manuscript. EP provided assistance and guidance in the data collection and analysis stage as well as in revising the manuscript.

## Supplementary files

Supplementary file 1. Official Information Request Template.

Supplementary file 2. Consulting Costs Raw Data.
