# Supplementary material for: Transparency in Healthcare Reporting: The Case of External Contractors and Consultants in New Zealand’s Healthcare System
Source: Int J Health Policy Manag. 2021 Jul 7;11(9):1642–9. doi: 10.34172/ijhpm.2021.69 (PMC9808226; doi:10.34172/ijhpm.2021.69)
Supplement: Supplementary file 1 — Official Information Request Template. [file ijhpm-11-1642-s001.pdf]

Article title: Transparency in Healthcare Reporting: The Case of External Contractors and Consultants in New Zealand's Healthcare System

Journal name: International Journal of Health Policy and Management (IJHPM)

Authors' information: Adeel Akmal[1]*, Robin Gauld[1*], Erin Penno[2]

[1]Centre for Health Systems and Technology, Otago Business School, University of Otago, Dunedin, New Zealand.

[2]Department of Preventive and Social Medicine, University of Otago, Dunedin, New Zealand.

(*Corresponding authors: adeel.akmal@otago.ac.nz & robin.gauld@otago.ac.nz)

Supplementary file 1. Official Information Request Template

Dear [DHB Person]

We are writing to you to file an official information request with your DHB organisation. The information we are seeking is related to external consultants—parties external to your organisation contracted to provide advice, expertise or training to any one or multiple departments or directorates in your DHB organisation.

1. Please provide the details of all external consultancies engaged over the last four years (from 01 July 2016 to 30 June 2019) which cost in excess of $10,000 to the DHB, i.e., a total of $10,000 or more were paid to the consultants for the project. We would appreciate it if the following information could be provided in an electronic spreadsheet format (MS Excel):
    1.1. The year in which the consultancy was engaged.
    1.2. The name of the organisation engaged to carry out the consultancy.
    1.3. The purpose of the consultancy or the type of service provided.
    1.4. The departments or directorates directly involved in the consultancy project.
    1.5. The cost of the consultancy.
    1.6. Whether or not tenders were invited.
2. What was the total amount spent on external consultants across all categories in each year?
3. Do you have a current policy on the use of external consultants? If yes, please provide a copy of this.
4. Do you have a current policy in place for determining whether external consultancies provide value for money? If yes, please provide a copy of this.

Note: We have submitted official information requests to all the New Zealand DHBs. This information will be used by a team of University of Otago researchers; and won't be shared with anyone other than the research team. All the DHB names and their information will be fully anonymized, and hence, no named reference to any particular DHB will be made in the final publication and any (or all) the subsequent press releases made in relation to the publication.
